# Varicocele

**Published:** 1859-04

**Authors:** 


*Varicocele—Clinical Lecture of M. Nélaton.*—Messrs. Editors: Some of your readers may be pleased to know the views of this surgeon, who, having been attached for many years to the Military School of St. Cyr, was enabled to examine several cases of varicocele, and was induced to believe that this affection, though by no means rare, is neither well understood nor suitably treated—that errors are found in all the surgical works mentioning the subject—that the general causes to which its formation is attributed are wrongly stated, and really have no bearing in the matter.

Among the causes which our classical writers have much insisted on are found the three following:—hernia, with its consequent treatment, the truss; abdominal tumors; constipation.

If one examines the period of life when the varicocele is most frequently seen, namely, from the sixteenth to the twentieth year, instantly he has a negation of the causes mentioned above, and considered the more predisposing agents in the malady.

Firstly: Hernia is very rare at that age. M. Malgaigne, in 300 cases occurring between the ages of ten and forty years, finds only twenty-six cases between the tenth and twentieth year.

Secondly: Abdominal tumors are excessively rare in young subjects, especially at that period when you encounter the varicocele.

Thirdly: Constipation. Young subjects are but rarely found who labor under this affection to a degree which, by its obstinacy, could be sufficient to produce a compression on the spermatic vein, and form the varicocele.

Again, hernia is much more frequent at the right than the left side—whereas varicocele is found almost constantly at the left.

From the autopsies which M. Nélaton has made, he proves that when a varicocele exists, the spermatic vein is tortuous, knotted and dilated throughout its course in the abdominal cavity; the hernia sac or the truss pressing upon the vein would cause the dilatation of the vessel below the inguinal ring only, and not within the cavity of the abdomen.

Anatomy has furnished a *supposed* solution of this abnormal condition, and to the question, why is the varicocele most frequently found in the left spermatic vein, has given a plausible explanation, by referring to the anatomical disposition of the vein, and the manner in which it joins the large trunk into which it pours its contents.

The right spermatic vein, near its junction with the ascending vena cava, pursues a direction nearly similar to that of the larger vessel, and by a gradual approach joins it an acute angle, the two currents readily uniting and flowing onward without obstruction.

The left, on the contrary, it is stated, joins the emulgent renal vein at a right angle, thus in a direction perpendicular to the current of blood coming from the kidney—a current considerably larger and moving with greater force. From this it appears that the spermatic vein is unable to empty its contents into the renal, in consequence cf which, is formed the varicocele.

This, however, is not true; the left spermatic vein does not enter the renal vein in a direction perpendicular to the latter, but bending outward from its course, turns again inward, describing a double curve on itself, and falls into the renal vein, forming an acute angle, as the right spermatic in its junction with the vena cava.

Another reason assigned for the frequency of varicocele in the left spermatic vein is its greater proportional length. This may be disproved by the fact that a varicosed condition of the spermatic is not more common in tall men than in those of medium stature, though naturally we should find the veins longer in the former class.

The evil consequences of varicocele have been much overrated. Many authors state an atrophy of the testicle follows the varicosed condition of the vein. This is not by any means proved. To judge properly of the question, one should have ascertained that the subject was endowed with equal health and strength in each testicle before the appearance of the varix, and that after its advent, the testicle had diminished.

That you find the testicle smaller when a varicocele exists, is at times true. But this is owing neither to a diminution in the testicle, nor an arrest in its development; the fact that the gland is small here, does not depend on the preëxistence of the varicocele, but they coëxist accidentally. Nor because the testicle is small, can you judge that its power of secretion is less than its fellow gland; not unfrequently will you find a considerable difference in the weight of these glands, though their mutual functions are equally performed.

M. Nélaton thinks varicocele an affection whose cause is unknown—usually found in youth, and rare in old age—that it disappears as man matures, and that the smaller ones are the most painful.

His treatment is determined by the facts, that they generally exist without pain, do not cause much inconvenience, that they *do not* cause an atrophy of the testicle, nor any loss of its power, and that they disappear with maturity. He therefore insists on a palliative treatment—in ordinary cases, the use of a suspensory bandage; when considerable inconvenience arises, you may swathe the scrotum, thus supporting and compressing moderately the vessel, similarly to the elastic stocking for varicose veins of the leg—and only operating, as the last measure in those cases where the pain is insupportable. Yours, with respect,

Paris, January 29, 1859. HALL CURTIS.

—*Boston Medical and Surgical Journal.*

